# The effects of type I collagenase on the degelification of chimpanzee (*Pan troglodytes*) semen plug and sperm quality

**DOI:** 10.1186/s12917-018-1389-0

**Published:** 2018-02-27

**Authors:** Jane-Fang Yu, Yu-Hua Lai, Tse-En Wang, Yu-Syuan Wei, Yu-Jia Chang, Sheng-Hsiang Li, Shih-Chien Chin, Radhika Joshi, Hui-Wen Chang, Pei-Shiue Tsai

**Affiliations:** 1 Conservation and Research Center, Taipei Zoo, 30 Xinguang Road, Section 2, Wenshan, Taipei, 11656 Taiwan; 2 Department of Veterinary Medicine, National Taiwan University, No. 1, Sec. 4, Roosevelt Rd, 10617 Taipei, Taiwan; 3 Graduate Institute of Veterinary Medicine, National Taiwan University, No. 1, Sec. 4, Roosevelt Rd, 10617 Taipei, Taiwan; 4 Research Center for Developmental Biology and Regenerative Medicine, National Taiwan University, No. 1, Sec. 4, Roosevelt Rd, 10617 Taipei, Taiwan; 5 Graduate Institute of Molecular and Comparative Pathobiology, National Taiwan University, No. 1, Sec. 4, Roosevelt Rd, 10617 Taipei, Taiwan; 6 Department of Medical Research, Mackay Memorial Hospital, No. 92, Section 2, Zhongshan N. Rd, 251 Tamshui, Taiwan

**Keywords:** Collagenase, Sperm preservation, Genetic diversity, Chimpanzee

## Abstract

**Background:**

Semen from the chimpanzee species becomes a colloidal solid after ejaculation. The formation of this copulatory plug is believed to prevent additional spermatozoa of subsequent mating events from accessing the ova. However, this naturally preserved strategy hampers the processes for sperm preparation. In this study, we investigated whether collagenase can be used to degelify the semen plug and accelerate the semen liquefaction process in zoo captive chimpanzee species (*Pan troglodytes*).

**Results:**

We showed that incubation of chimpanzee ejaculates with 0.1% type I collagenase efficiently and significantly (*p* < 0.05) releases 2.7-fold more spermatozoa from the coagulated ejaculates, and this degelification process did not alter sperm morphology or viability; nor did it stimulate spontaneous capacitation or an acrosome reaction as assessed by tyrosine phosphorylation and peanut agglutinin stains; moreover, based on computer assisted sperm analysis assay, motility-related parameters remained similar to those of untreated spermatozoa. When collagenase effects were evaluated on cryopreserved sperm samples, we observed post collagenase treatment in which 2.5% glycerol, as a cryoprotectant, preserved sperm acrosome integrity better than 7.8%; however, 7.8% glycerol, as a cryoprotectant, maintained sperm motility better than that of 2.5% glycerol.

**Conclusions:**

Our results demonstrated for the first time that type I collagenase can be used to obtain a significantly higher number of spermatozoa from colloid chimpanzee semen ejaculate without affecting the physiological properties of spermatozoa, and these results are critical for the subsequent gamete development. Our results would benefit sperm preparation processes and cryopreservation efficiency per ejaculate, as more unaffected spermatozoa can be released from the semen plug within a shorter period of time. These results would also benefit the genetic diversity of the chimpanzee species, using sperm cells from less dominant individuals, and for achieving better pregnancy success in primates with significantly higher amounts of sperm for artificial insemination.

**Electronic supplementary material:**

The online version of this article (10.1186/s12917-018-1389-0) contains supplementary material, which is available to authorized users.

## Background

Seminal fluid is a mixture of various components secreted by gonads and accessory sex glands that contains proteins, lipids, amino acids, fructose and other carbohydrates. In addition to the vitality nutrients and metabolites mentioned above, seminal fluid also contains numerous components that form the extracellular matrix and contributes to the main composition of semen and its viscosity, which further affects sperm competition success [1–4]. The degree of semen viscosity varies between animal species and individuals. Ejaculates from some animals remain liquid all the time, while semen from other mammalian species becomes solidified after ejaculation. It is known that semen from humans (*Homo sapiens*) undergoes solidification and liquefaction processes [5]; however, semen from other species, such as llamas, alpacas or primates (i.e., chimpanzee, orangutan, gorilla), the ejaculates begin to coagulate and become a colloidal solid after exposure to the air [6, 7]. The formation of this so called “mating plug” or copulatory plug is believed to prevent the additional spermatozoa of subsequent mating events from accessing the ova, which is critical to preserve genetic materials from a specific individual (especially the dominant individual from a species with highly hierarchical social structure) in wild animals [8].

It has been suggested that vesiculase secreted from anterior prostate or coagulating gland facilitates the coagulation of the semen and proteolytic enzymes in human seminal fluid are responsible for semen liquefaction, likely due to high levels of arginine ester-hydrolyzing enzyme [5]. Earlier studies on semen composition of primates indicated that their semen may not contain sufficient amounts of proteolytic enzymes to rapidly and automatically liquefy colloidal semen plug [5, 8]; therefore, current approach to obtain free swimming spermatozoa from great apes is to place the ejaculates at either room temperature (RT) or at 25–37 °C and wait for auto-liquefaction. However, Young and Smithwick demonstrated that in chimpanzee *(Pan troglodytes*) ejaculates, only about one-fourth of the ejaculates were automatically liquefied after 4 h, and sperm motility measured during this 4 h period of time showed a significant downward tendency, especially in the first two, one-hour (0 ~ 1 h and 1 ~ 2 h) intervals. Additionally, the percentage of viable and motile spermatozoa decreased significantly over time [9]. In this study, we aim to investigate whether type I collagenase would facilitate semen liquefaction of chimpanzee semen and to evaluate whether collagenase treatment would affect sperm capacitation status, acrosome integrity and motility-related parameters using various independent approaches.

## Methods

### Chimpanzee training and semen collection by hand massage

Electroejaculation (EEJ) is widely used for semen collection in wildlife and captive zoo animals [10, 11]. However, EEJ in great apes has been discouraged, as it incurs higher risk due to the need for deep anesthetization and the high electric voltage required to induce ejaculation. Moreover, the quality of ejaculates was generally suboptimal with great variation. To perform non-invasive semen collection procedure, as previously described [12, 13], Taipei Zoo began to train great apes for medical, husbandry and research purposes using positive reinforcement methods (e.g., rewarding additional feed or biscuits) since 2010 (Additional file 1: Figure S1). Chimpanzees were trained to stay at their assigned position in their accommodation facing animal caretakers when being called. Chimpanzee displayed an erect penis ready for semen collection when veterinarians approached with artificial vagina in hand (Additional file 1: Figure S1A). Other chimpanzees in the same cage held still at their position with the animal caretaker and did not interfere with semen collection processes (Additional file 1: Figure S1B). Ejaculate was immediately collected from the artificial vagina into a 50 ml falcon tube using a sterilized scrapper in the presence of dilution medium; the formation of gel-like texture was observed as soon as the ejaculate got in contact with the air (Additional file 1: Figure S1C). As indicated in the methods (animal training section), after the completion of semen collection, the chimpanzee was given additional rewards (biscuits) on top of their regular feeds (Additional file 1: Figure S1D).

Semen samples acquired in this study were collected from 4 adult chimpanzees (*Pan troglodytes*, age ranging from 7 to 23 years old, group housed with fixed feeding schedule and free water access) using artificial vagina (IMV Ram/goat vagina 13.5, IMV Technologies, L’Aigle, France) in combination with penile hand massage without anesthesia.

### Chemicals, reagents, antibodies

Chemicals and reagents were obtained from Sigma-Aldrich (St. Louis, MO, USA) unless otherwise stated. Mouse monoclonal anti-Phosphorylation Tyrosine (4G10) was obtained from EMD Millipore (Darmstadt, Germany); Lectin-PNA from *Arachis hypogaea* conjugated with Alexa 488 was purchased from Invitrogen (Invitrogen/Life Technologies, Carlsbad, CA, USA). All secondary antibodies were obtained from Jackson ImmunoResearch (PA, USA). Sperm incubation medium (Modified Ham’s F10) and frozen medium used for cryopreservation experiments were acquired from IrvineScientific (CA, USA). Type I collagenase from *Clostridium histolyticum* was purchased from Sigma-Aldrich.

### Semen evaluations and sperm preparation

The acquisition of animal materials (semen ejaculates) and animal handling followed the regulation and approval of animal welfare committee of Taipei Zoo (protocol#10601) and were monitored under the guidance of the animal welfare committee of Taipei Zoo, Taiwan. For sperm preparation, 16 ejaculates (4 ejaculations each from 4 individuals) were collected and assessed (Additional file 2: Figure S2). Freshly ejaculated semen was removed from the artificial vagina into a 50 ml falcon tube using sterilized chemical scope. An equivalent amount (in weight) of clotted semen was separated for further experiments. Experiments were divided into “fresh sample evaluations” and “cryopreserved sample assessments”. For fresh sample experiments, coagulated semen was incubated with Ham’s F10 medium in the absence (control) or presence of 0.1 type I collagenase at 25 °C water bath for 30 min. After the incubation, liquefied semen was separated for semen quality assessments; semen concentration, motility, sperm viability and morphological abnormality were manually evaluated by experienced personnel. For morphological evaluation, standard papanicolaou (PAP) and POPE’s staining procedures were carried out as previously described [14–16], and 200 sperm cells were evaluated for each collection; data were expressed in percentages (%). For cryopreservation experiments after required collagenase incubation (with or without collagenase treatment), spermatozoa were spun down (800 g, 10 min) and resuspended in a sufficient volume of frozen medium containing test yolk (IrvineScientific) to achieve a final concentration of ~ 10^9^ spermatozoa/ml. Sperm-test yolk mixture was first cooled down at 25 °C in a water bath for 30 min at dark and then chilled in a cooler at 4 °C for 90 min (cooling rate of < 0.5 °C/min), and chilled sperm samples were mixed with 12% glycerol (in test yolk) to reach a final concentration of either 2.5% or 7.8% glycerol before loading into plastic straws (0.25 ml) at 4 °C. Chilled straws containing semen samples were subsequently exposed to nitrogen vapor (4 cm over the surface of liquid nitrogen) for 10 min and then immersed and stored in liquid nitrogen until use.

### Sperm viability assay

Sperm 3-(4,5-dimethyl thiazol-2-yl)-2,5-diphenyl tetrazolium bromide (MTT) assay and Propidium iodine (PI) staining were performed to evaluate sperm viability. Briefly, MTT stock (5 mg/ml) solution was prepared and filtered with 0.22 μm PVDF membrane filter (Millipore) to remove undissolved residues and kept at 4 °C in dark for maximum 1 week. When used, MTT stock was added into sperm suspensions to a final concentration of 0.5 mg/ml (1.2 mM) in Ham’s F10 medium and incubated for 1 h at 37 °C in humidified incubator. Analysis was carried out by manually examining at least 200 sperm cells per condition; spermatozoa without any signs of residue MTT were considered non-viable sperm; spermatozoa with clear granule-like particle aggregated in the mid-piece region were considered viable spermatozoa [17]. Due to the interference of cryoprotectant on MTT assay, PI stain was therefore used for cryopreserved sample evaluation as an alternative measurement as previously described [18]. In brief, after frozen straws were defrosted in a 37 °C pre-warmed water bath, the frozen medium was removed by centrifugation (800 g, 10 min), and sperm pellet was resuspended in Ham’s F-10 medium. A half microliter of stock PI (1 mg/ml) was added to each sample, obtaining a final concentration of 5 μg/ml (7.5 μM). Samples were incubated at 37 °C in a dark room for 8 min before manual assessments under fluorescence microscopy.

### Computer-assisted sperm analysis (CASA)

Motility-related sperm parameters were analyzed using an UltiMate computer-assisted sperm analysis system (CASA, Hamilton Thorne Inc., Beverly, USA), as described by Broekhuijse et al. [19]. Default software and parameter settings were followed by recommendation of Hamilton Thorne Inc., for human spermatozoa, and image capture was set to 60 frames/s; a total 45 frames were recorded per examination field. After required treatments, sperm suspension was diluted 1:10 in pre-warmed Ham’s F10 medium. Then, 3 μl aliquot was added into a standardized Leja 4-chamber counting slide (Leja Products B.V., Nieuw Vennep, the Netherlands). By the use of automated stage, 5 microscopic fields were analyzed within each chamber (a total 225 frames were taken per experimental sample). At least 3–5 independent repeats were performed for each experimental condition; mean value and standard error of mean (SEM) were calculated accordingly. Motility-related parameters including motility (%), progressive motility (%, defined as VAP > =25 μm/s, STR > =30%), velocity average path (VAP, μm/s), velocity straight line (VSL, μm/s), velocity curvilinear (VCL, μm/s), amplitude of lateral head displacement (ALH, μm), beat frequency cross (BCF, Hz), and straightness (STR) were measured and analyzed.

### Indirect immunofluorescent (IFA) assays for sperm capacitation status and acrosome integrity

Indirect immunofluorescent staining was carried out to evaluate sperm capacitation status and acrosome integrity. For sperm capacitation status, standard protocol for sperm phosphotyrosine staining was followed [20]. Spermatozoa were fixed with 4% paraformaldehyde (PFA) for 30 min at RT, permeabilization of fixed spermatozoa was performed by using 0.1% (*v*/v) Triton X-100 in PBS for 10 min at RT. Tyrosine phosphoproteins were labelled and subsequently recognized by anti-mouse secondary antibody. Fully capacitated spermatozoon was defined when either (1) a strong signal could be observed in the flagellum or (2) a strong signal in the flagellum with moderate signal in the equatorial segment was detected. Acrosome integrity was evaluated as previously described [21]. Briefly, fixed spermatozoa were washed and resuspended in PBS; 20 μl of sperm suspension was added onto Superfrost Plus Microscope Slides (Thermo Scientific/Invitrogen), and spermatozoa were allowed to attach to the slide for 10 min. Unbound sperm cells were removed with PBS, and antibody incubation was immediately carried out using peanut agglutinin (PNA) conjugated with Alexa 488 at 2 mM for 1 h at RT. Nuclei were stained with 4′,6-diamidino-2-phenylindole (DAPI) (Vectashield H-1200, Vector Laboratories, CA, USA), and slides were then sealed with nail polish. At least 200 spermatozoa were counted in each sample under a fluorescence microscope. For negative controls, each immunoreaction was accompanied by a reaction omitting the primary antibody. All samples were evaluated with Olympus IX83 epifluorescence microscopy (Olympus, Tokyo, JP) and analyzed with either ImageJ (NIH; http://rsb.info.nih.gov/ij/) or Cellsens software (Olympus).

#### Immunoblotting

Sperm cells were solubilized using commercially available RIPA cell lysis buffer (AMRESCO, Ohio, USA) in combination with sonication. Equivalent amount of total protein extract was resuspended with an appropriate volume of Lithium dodecyl sulfate (LDS) loading buffer (Invitrogen) in the presence of reducing agent (50 mM DTT). Samples were heated in a 100 °C dry bath for 10 min and cooled on ice before loading on gels. Proteins were separated by SDS-PAGE (gradient T-Pro EZ Gel Solution, T-Pro Biotechnology, NTC, TW) and wet-blotted onto a PVDF membrane (Immobilon-P, Millipore, Billerica, MA, USA). After blocking for 1 h with blocking buffer (5 mM Tris, 250 mM sucrose, pH 7.4 with 0.05% *v*/v Tween-20 [TBST], supplemented with 5% milk powder) at RT, blots were incubated with anti-Phosphotyrosine (Merk/Millipore) primary antibody and subsequently with secondary antibody (1:5000 dilution in TBST) for 1 h at RT. Protein signals were visualized by chemiluminescence (Merck, Ltd., TW) and were detected with ChemiDoc™ XRS+ system (Bio-Rad).

#### Statistical analyses

Results were expressed as the mean+/−standard error of mean (SEM). Comparative studies of means were performed using a one-way analysis of variance (ANOVA) followed by a Kruskal- Wallis test. Significance was set with *p* < 0.05.

## Results

### Type I collagenase facilitated the release of spermatozoa from colloid semen without affecting sperm morphology and viability

**Fig. 1:**
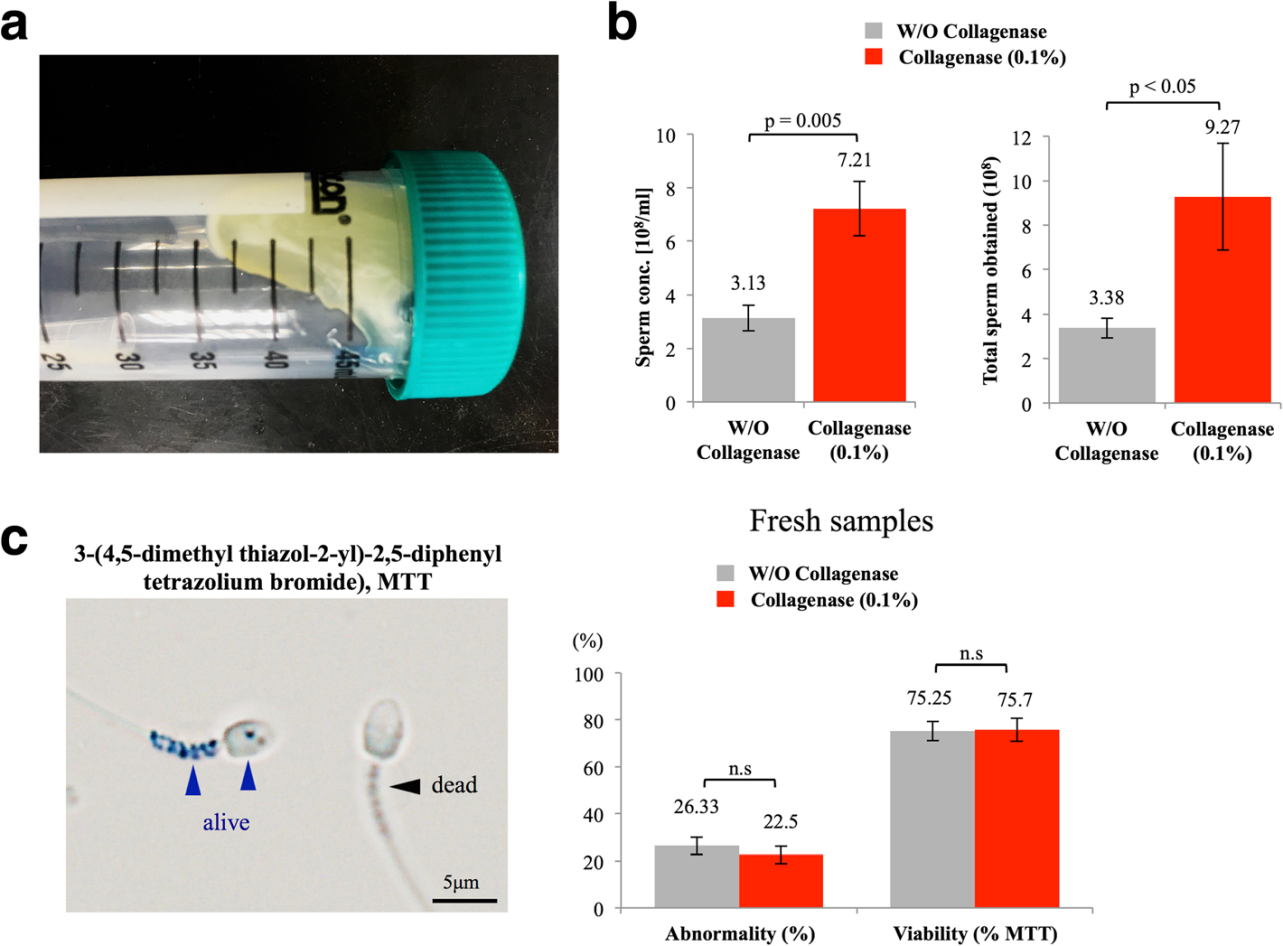
Type I collagenase facilitated the release of spermatozoa from colloid semen without affecting sperm morphology and viability. **a** Chimpanzee semen formed colloid structure, exhibited high viscosity. **b** 2.4-fold increase in sperm concentration per ml was achieved, and approximately 2.7-fold more spermatozoa in number were collected when type I collagenase was used. **c** No significant differences were detected on sperm abnormality or sperm viability between control and collagenase-treated groups; W/O: without; MTT: 3-(4,5-dimethyl thiazol-2-yl)-2,5-diphenyl tetrazolium bromide. At least 200 sperm cells were evaluated in each sample. Data were analyzed from 16 ejaculates from 4 individuals

In line with observations from earlier studies [10, 22], ejaculations from chimpanzees formed a colloid semen texture as soon as they were exposed to the air, and only a minimal amount of semen in liquid form could be observed (Fig. 1a). An average concentration of 3.13 × 10^8^ sperm/ml could be obtained from auto-liquefied semen, in contrast, a 2.3-fold increase in sperm concentration per ml (7.21 × 10^8^/ml) was achieved in the presence of 0.1% collagenase, and a 2.74-fold (3.38 × 10^8^ vs. 9.27 × 10^8^) increase in total sperm number was counted (Fig. 1b). To evaluate the cell toxicity of collagenase and whether collagenase treatment results in sperm death, MTT sperm viability assay was performed as described earlier [17]. Spermatozoon was considered alive when positive signals (granule-like blue particles, Fig. 1c, indicated with blue arrow heads) were observed at its mid-piece, or else the spermatozoon would be considered as dead (Fig. 1c, indicated with black arrow head). For sperm abnormality assessment, chimpanzee sperm cells were smeared on examination slides and manually evaluated by 3 independent experienced lab technicians. No significant differences were observed between control and collagenase-treated groups in sperm abnormality rate, nor did their viability suggest that collagenase treatment did not cause morphological damages or affect chimpanzee viability under our experimental setups (Fig. 1c).

### Motility-related sperm parameters were not altered by type I collagenase treatment

**Fig. 2:**
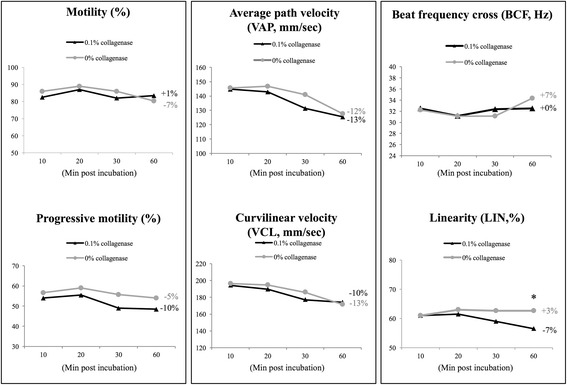
CASA analyses revealed no negative effects of type I collagenase on sperm motility-related parameters. Computer-assisted sperm analysis showed no significant differences on sperm motility, progressive motility, average path velocity (VAP), curvilinear velocity (VCL), beat frequency cross (BCF), and linearity (LIN) between control and collagenase-treated sperm, indicated no negative effects of type I collagenase on sperm motility-related parameters. Data were expressed in percentages (%, for motility, progressive motility and linearity), mm/s (for VCL) and Hz (for BCF). *Indicates significant differences between groups. Relative changes within each group (t60 vs. t10) were also indicated. Data were analyzed from 16 ejaculates from 4 individuals

Sperm motility is the most important factor for successful fertilization in vivo; we next evaluated the effects of type I collagenase on sperm motility using a well-recognized system, namely, computer-assisted sperm analysis (CASA). When compared with control condition, chimpanzee sperm treated with collagenase did not show significant decrease in total motility; when compared with its own starting point (t = 10), gradual reductions in progressive motility were measured in both control and collagenase-treated groups (Fig. 2). Among all parameters measured, average path velocity (VAP) and curvilinear velocity (VCL) were widely used to indicate the velocity/speed of swimming sperm cells; beat frequency cross (BCF) and linearity (LIN) are known to represent sperm swimming pattern, and these four parameters were considered critical indicators for sperm functions measured by CASA [19]. Our data demonstrated that besides a slight decrease in sperm swimming linearity in the collagenase-treated group at 60 min post incubation, and no other differences were measured between control and collagenase-treated groups in the abovementioned parameters (Fig. 2); therefore, our data showed that collagenase did not exhibit apparent negative influences on sperm swimming and motility-related parameters in freshly collected chimpanzee semen samples.

### Type I collagenase did not induce spontaneous sperm capacitation or acrosome reaction

**Fig. 3:**
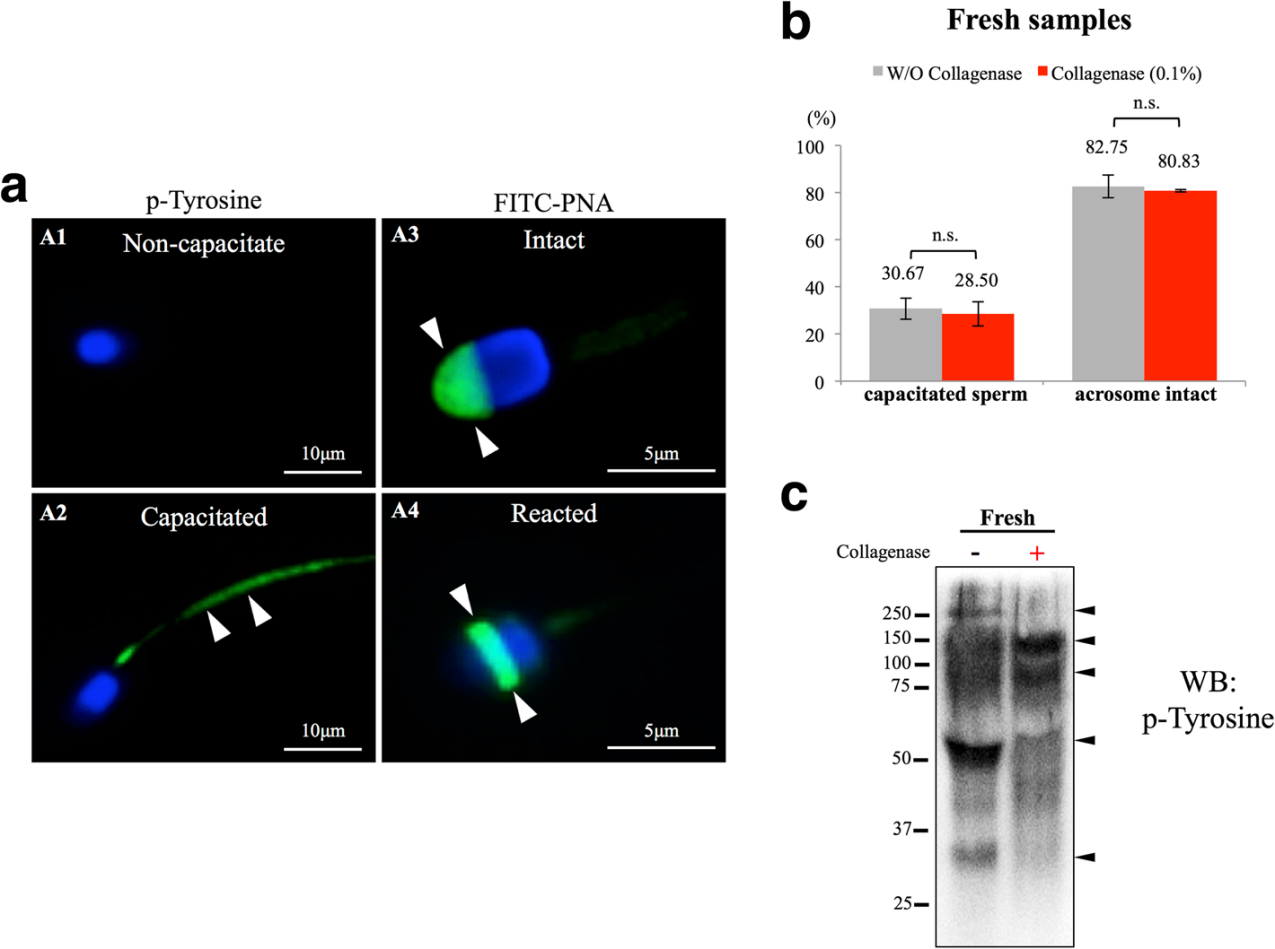
Type I collagenase treatment did not result in spontaneous capacitation or acrosome reaction. **A** Sperm capacitation and acrosome integrity accessed by tyrosine phosphorylated proteins and the presence of outer acrosome-specific lectin *peanut agglutinin* (PNA). Spermatozoon was considered capacitated when distinct tyrosine phosphorylation staining was observed at sperm tail (**A1**, **A2**, indicated with white arrow heads). Sperm acrosome was considered intact when homogeneous PNA staining could be observed at the anterior area of the sperm head (**A3**, marked with white arrow heads). Acrosome reacting/reacted spermatozoon showed a ring-like staining pattern at its post-equatorial region (**A4**, marked with arrow heads). **B-C** No significant differences on sperm capacitation and acrosome reaction rates were observed between control and 0.1% type I collagenase-treated group; N.S: not significantly different. At least 200 sperm cells were evaluated in each sample. Data were analyzed from 16 ejaculates from 4 individuals. Fifty micrograms of total protein was loaded onto each lane for immunoblotting experiment

Sperm capacitation and acrosome reaction are two important indications for physiological status of sperm cells [21, 23, 24]. Tyrosine phosphorylation is a well-recognized indicator for both spontaneous or induced sperm capacitation; spermatozoa were considered capacitated when tyrosine phosphorylated proteins can be observed at sperm tail (Fig. 3A2, indicated with arrow heads). For acrosome integrity, outer acrosomal membrane-specific lectin *peanut agglutinin* (PNA) was used as a marker lectin in this study as previously described [21]. Acrosome was considered intact when a homogeneous PNA signal can be observed at the anterior sperm head (Fig. 3A3, indicated with arrow heads) and acrosome reacted or reacting spermatozoa exhibit no or ring-like signal at their equatorial area of the sperm heads (Fig. 3A4, indicated with arrow heads). Following these criteria, at least 200 sperm cells were counted for each experimental repeat (*n* = 16); a slight decrease in sperm capacitation rate was observed when 0.1% collagenase was used (30.7% vs. 28.5%); however, this difference did not reach significance (*p* > 0.01, Fig. 3B). For spontaneous acrosome reaction rate, no significant difference was observed between control and collagenase-treated groups (p > 0.01, Fig. 3B). In addition to sperm staining mentioned above, we also assessed the level of tyrosine phosphorylation using immunoblotting technique. In line with our sperm counting results, both control and collagenase-treated conditions exhibited a certain level of tyrosine-phosphorylated proteins, but no apparent differences were detected between the two groups (Fig. 3C). Taken together, our data showed that type I collagenase did not alter sperm capacitation status or cause further acrosome damages on freshly acquired chimpanzee sperm cells.

### Collagenase-treated spermatozoa showed similar morphological and physical characteristics as un-treated spermatozoa after glycerol-based cryopreservation

**Fig. 4:**
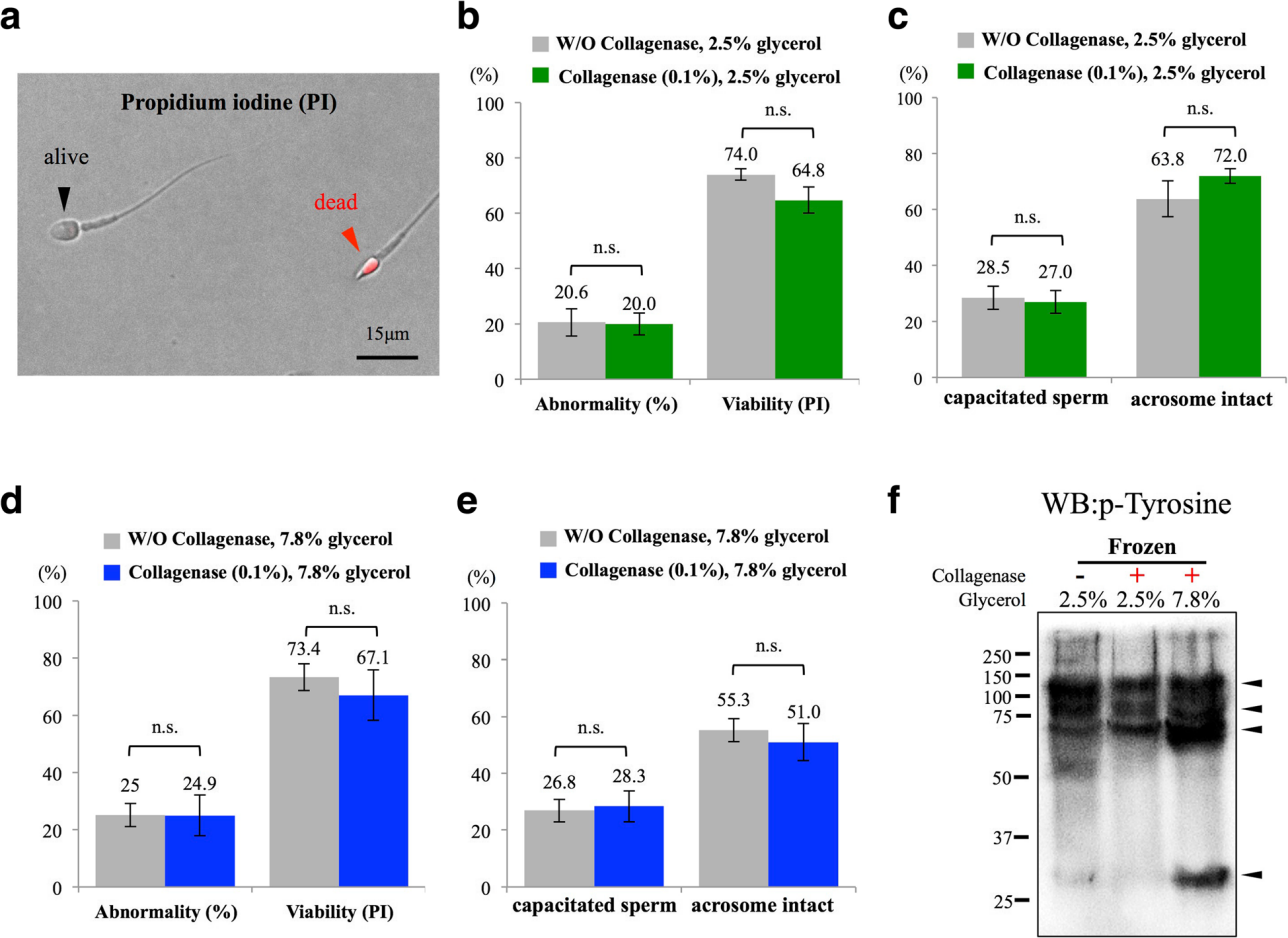
Analyses on cryopreserved chimpanzee spermatozoa revealed no differences between control and collagenase-treated groups. **a** Propidium iodine (PI) was used to quickly assess the viability of cryopreserved semen samples; alive sperm cells showed negative signal while dead sperm cells were stained in red (indicated with arrow heads). **b-d** Chimpanzee sperm cells treated with 0.1% collagenase showed no differences in sperm abnormality, viability, capacitation rate or acrosome integrity compared with control sperm cells after cryopreserved with either 2.5% glycerol (**b-c**) or 7.8% glycerol (D-E). **f** A slight increase in tyrosine phosphorylation was detected in collagenase-treated group when 7.8% glycerol was used for cryoprotectant. Arrowheads indicate tyrosine phosphorylated protein signals; N.S: not significantly different. At least 200 sperm cells were evaluated in each sample. Data were analyzed from 16 ejaculates from 4 individuals

Glycerol has been considered as an affective cryoprotectant; based on current practice in most of the zoos, 2.5–7.8% glycerol was commonly used to preserve chimpanzee sperm [25], therefore, 2.5% and 7.8% glycerol were used in this study. Based on propidium iodine staining (Fig. 4a), no matter whether 2.5% or 7.8% glycerol was used as cryoprotectant, no differences were observed between control and collagenase-treated groups on morphological abnormality, viability, sperm capacitation status and acrosome integrity (Fig. 4b-e). From immunoblotting results, no apparent differences between collagenase-treated and untreated groups were observed when 2.5% glycerol was applied; however, a slight increase in tyrosine phosphorylated proteins was observed when 7.8% glycerol was used (Fig. 4f).

**Fig. 5:**
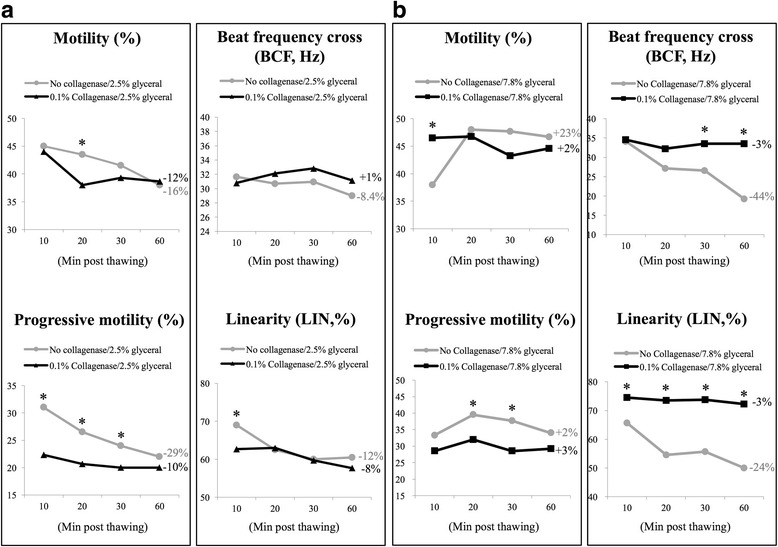
Collagenase-treated sperm cells exhibited relatively stable motility, progressive motility, beat frequency cross and linearity within 1 h post thaw. **a** In contrast to control sperm cells (in gray)**,** 2.5% glycerol cryopreserved and collagenase-treated chimpanzee spermatozoa (in black) exhibited a sharp decrease in motility at first 20 min. Similar to motility, control sperm showed a gradual decrease in progressive motility (− 29%); however, collagenase-treated sperm displayed a relatively static pattern throughout the time. **b** Under 7.8% glycerol preservation, collagenase-treated group (in black) showed a relatively stable pattern throughout the time when compared with control group (in gray). *Indicates significant differences between groups. Relative changes within each group (t60 vs. t10) were also indicated. Data were analyzed from 16 ejaculates from 4 individuals

When CASA was used for evaluating motility-related parameters of cryopreserved semen samples, some differences between control and collagenase-treated group were observed. In the 2.5% glycerol group (Fig. 5a), in contrast to the control group, spermatozoa showed a gradual decrease throughout time, and collagenase-treated spermatozoa showed a sharp decline in motility within the first 20 min post thaw and then became stable afterward (Fig. 5a). Collagenase-treated group showed a lower progressive motility throughout the measured time points when compared with control non-treated sperm cells; however, the level of reduction in progressive motility was more apparent in control non-treated spermatozoa when compared with collagenase-treated spermatozoa (− 29% vs. -10% for control non-treated and collagenase-treated group, respectively) (Fig. 5a). Intriguingly, both VAP, VCL were sharply decreased in the control group, while a static (VAP) and even a slight increase in VCL was observed in collagenase-treated group (data not shown). For sperm swimming pattern, when compared with control sperm when 2.5% glycerol was used, collagenase-treated spermatozoa showed a significant increase in BCF with similar linearity (Fig. 5a). When 7.8% glycerol was used for cryopreservation protocol (Fig. 5b), in contrast to the control group, non-treated spermatozoa exhibited greater variations throughout the measuring time points (0–60 min after thaw), and parameters, such as motility, progressive motility, BCF and LIN measured for collagenase-treated sperm, were relatively stable (Fig. 5b, compared gray lines with black lines throughout the time points).

### Glycerol concentrations used as cryoprotectant benefit for different aspects of chimpanzee spermatozoa

**Fig. 6:**
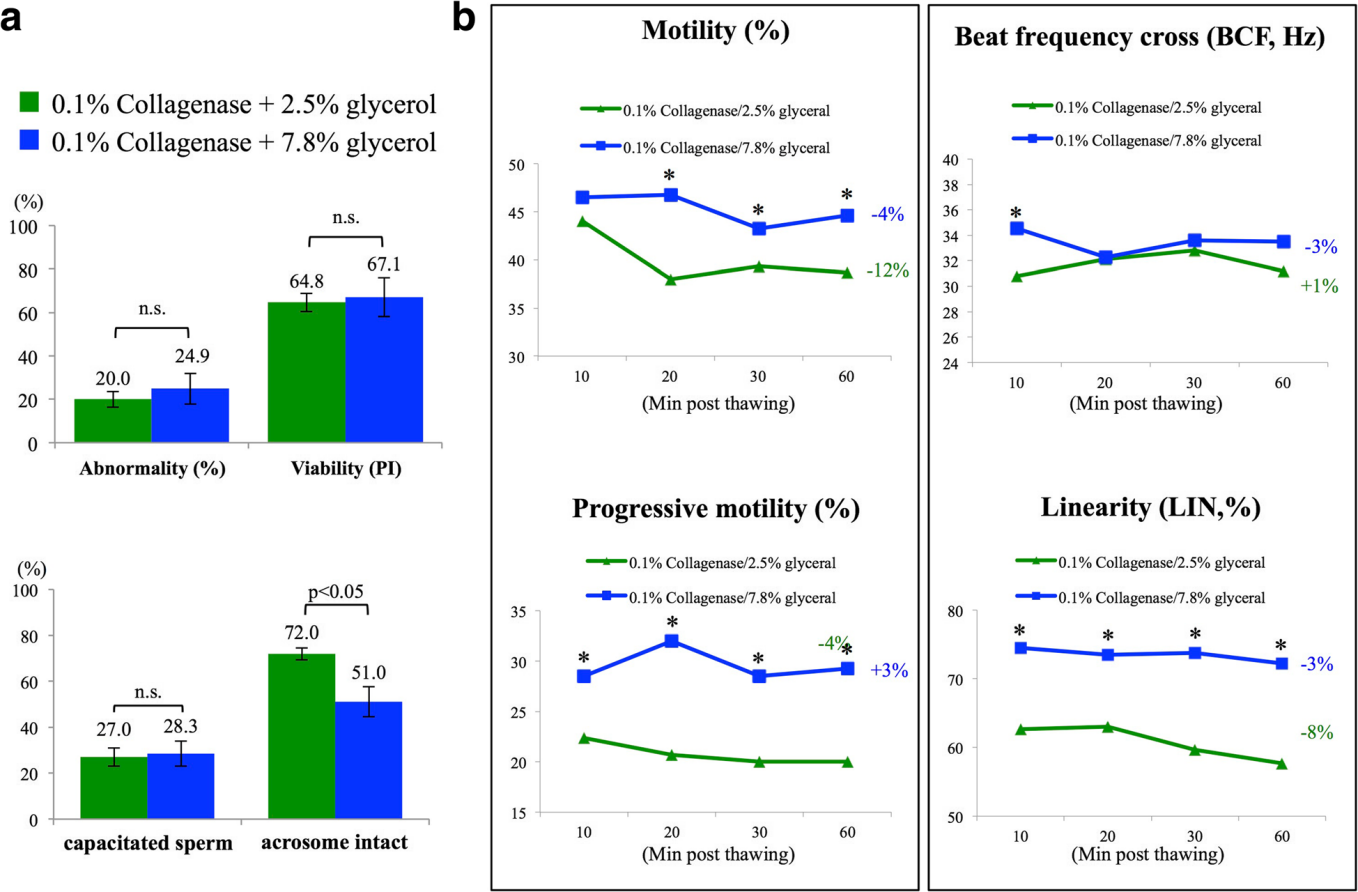
Glycerol concentrations resulted in different protective effects on either sperm membrane integrity or motility-related characteristics. **a** Collagenase-treated sperm cryopreserved with 7.8% glycerol showed higher abnormality rate and lower viability when compared with 2.5% glycerol cryopreserved group. Differences were also observed on acrosome integrity that 7.8% glycerol group contained significantly lower acrosome intact spermatozoa after cryopreservation processes (72% vs. 51% for 2.5% and 7.8% glycerol group). **b** Collagenase-treated chimpanzee sperm cryopreserved with 7.8% glycerol exhibited higher motility, progressive motility, beat frequency cross (BCF) and linearity (LIN) when compared with 2.5% glycerol group; N.S: not significantly different. At least 200 sperm cells were evaluated in each sample. *Indicates significant differences between groups. Relative changes within each group (t60 vs. t10) were also indicated. Data were analyzed from 16 ejaculates from 4 individuals

As cryopreservation processes normally compromise sperm motility and reduce fertilization success, we further compared whether different percentages of glycerol, as a cryoprotectant, could benefit cryopreservation outcomes, as measured by sperm morphology, viability, capacitation status, acrosome integrity and CASA analyses. With our cryopreservation protocol, when compared with 2.5% glycerol group, 7.8% glycerol group showed slightly higher abnormality rate and lower viability rate (Fig. 6a). Although no differences in spontaneous sperm capacitation rate were observed between 2.5% and 7.8% glycerol cryopreserved groups, the 2.5% glycerol cryopreserved group showed significantly higher percentage of acrosome intact spermatozoa after freezing-thawing process when compared with 7.8% glycerol cryopreserved group (72% vs. 51% for 2.5% vs. 7.8% glycerol) (Fig. 6a, lower panel). For sperm motility-related parameters, when 7.8% glycerol was used for cryopreservation, spermatozoa showed better motility, higher progressive motility, BCF and linearity, as measured by CASA analyses (Fig. 6b). These data demonstrated that both 2.5% and 7.8% glycerol could be used when freezing spermatozoa of chimpanzee sperm (*Pan troglodytes*), and glycerol concentration would be beneficial to different aspects of sperm physiological characteristics.

## Discussion

Reproduction of wildlife and zoo captive species is always a challenging issue due to low number of individuals and limited area of habitats. These reasons often result in the aging of the animals or close inbreeding within herds, which further affects reproductive success and leads to the extinction of specific species. Primate species, such as chimpanzees, have a strict social structure of hierarchy in their natural state, and therefore, in an ethnic group, only one dominant individual can proceed the breeding with females, thus greatly reduces genetic diversity in the particular population [26, 27]. In most species, this dilemma can be resolved by artificial insemination using semen samples collected from other non-dominant male individuals.

Unlike most of the animal species, semen samples collected from great apes (no matter by EEJ or hand message) display high viscosity and appear as gel-like structure with slow semen liquefaction in nature [8–10]. This characterization has also been observed in South American camelidae, such as llamas and alpaca, and likely serves to ensure the success of mating competition and the consequent inheritance of genetic materials [6, 10, 28, 29]. Upon waiting for the occurrence of natural semen liquefaction, sperm motility declines quickly and often results in poor semen quality and a low success rate for pregnancy in females when used for artificial insemination. To overcome this naturally coagulated semen, attempts have been made in camelidae species to facilitate semen liquefaction using various enzymes [6, 30, 31]. Results from these independent studies indicated that 1 mg/ml collagenase affectively eliminated viscosity of 100% and 99% of the samples in llamas and alpaca, respectively, while fibrinolysin also seemed to be effective; however, in enzyme-treated llama or alpaca semen samples, besides the general sperm motility and viability (assessed by manual evaluation) that were presented, no further information was available [6, 30]. Authors concluded that among enzymes tested, collagenase exhibited the best degelification effect with minimal negative impacts on semen quality [30, 31]. Our results demonstrated that in line with previous reports in camelidae species, type I collagenase was effective in eliminating semen viscosity and that a 2.3-fold increase in sperm concentration and a 2.7-fold increase in total sperm number were achieved in collagenase-treated group; however, collagenase concentration used in this study was lower (0.1% [*W*/*V*]) than that in studies in South American camelids, likely due to different semen composition and the proportion of collagen present in the semen of different species. Artificial insemination using cryopreserved samples or semen obtained from elderly individuals normally requires a higher number of spermatozoa to achieve pregnancy success [32, 33]; therefore, significant increase in sperm concentration and total spermatozoa obtained after collagenase incubation would benefit the success of pregnancy and increase the number of cryopreserved semen samples (total number of frozen straws) per ejaculation.

Parameters commonly used at clinics for semen quality assessment consist of sperm count semen characteristics, sperm motility (can be evaluated by CASA), mortality (viability), and morphology [34]. Our results showed that using 0.1% collagenase to facilitate the release of spermatozoa from the colloid semen coagulates did not lead to morphological alteration, nor did it lead to a decrease in cell viability. Moreover, collagenase treatment did not stimulate spontaneous sperm capacitation or damage acrosome integrity or affect motility-related parameters. Collagenase is an enzyme that facilitates the breakdown of collagen, a component, which contributes to the viscosity and structure of seminal fluid [35, 36]. Based on currently available sperm and semen proteome data, depending on animal species and physiological condition of the individual, collagen presents in various amounts in seminal fluid, but not on sperm membrane surface [36–41]; therefore, under our experimental condition (0.1% collagenase, 30 min at 25 °C), the effects of collagenase likely were limited to de-structure the collagen in the extracellular matrix of chimpanzee semen and may not have directly affected membrane composition of chimpanzee spermatozoa, as the percentage of spontaneous capacitation or acrosome-reacted sperm did not increase under collagenase treatment when compared with its control conditions.

Cryopreservation of gametes not only preserves genetic materials but also promotes genetic diversity through semen/gamete exchange programs between zoos or by artificial insemination of females using semen samples collected from less dominate individuals. As we demonstrated in this study, collagenase treatment did not affect examined sperm parameters in freshly acquired semen samples. To further evaluate whether collagenase affects cryoprotective efficiency of glycerol-based cryopreservation protocols, we compared sperm capacitation status, acrosome integrity as well as sperm motility-related parameters based on post thaw semen samples of two glycerol concentrations (2.5%, 7.8%). No significant between-group differences could be observed, with or without collagenase, when either 2.5% or 7.8% glycerol was used for cryopreservation, although a slight increase of tyrosine phosphorylation was detected when immunoblotting technique was applied. This result further supported the fact that collagenase did not significantly affect physiological characteristics of chimpanzee spermatozoa, even if these treated sperm cells were subjected for cryopreservation. In contrast to an earlier report from Gould et al., which stated that 7.8% glycerol, as a cryoprotectant, resulted in better cryopreservation outcomes [25], we observed a sharp decrease in acrosome integrity when 7.8% glycerol was used. The observed inconsistencies may likely be due to different experimental setups as well as different freezing protocol and medium used; moreover, the defined “better cryopreservation outcomes” in earlier study by Gould et al. focused primarily on sperm motility rather than the additional parameters evaluated in this study.

Our data from CASA experiments demonstrated that at least in freshly acquired chimpanzee samples, parameters evaluated (motility, progressive motility, VAP, VCL, BCF and LIN) were not affected by collagenase treatment. However, in cryopreserved semen samples, a significant decrease in progressive motility was observed in collagenase-treated group when 2.5% glycerol was used. One interesting phenomenon was that in contrast to fast decline in motility, progressive motility, BCF and LIN in control sperm, collagenase-treated sperm cells showed relatively stable swimming patterns throughout time, as measured by CASA; further experiments will be required to reveal whether collagenase could also facilitate sperm cells in preserving energy due to its reaction on quickly dissolving extracellular matrix of highly viscous chimpanzee semen. In this study, we observed that after collagenase treatment, 7.8% glycerol resulted in better post-thaw motility, progressive motility BCF and LIN; this outcome can be explained by our observation that 7.8% glycerol group showed an increase of tyrosine phosphorylated proteins, which resulted in an increase of hypermotile capacitated sperm; however, 2.5% glycerol provided significantly better protective effect on sperm acrosome integrity than that of 7.8% glycerol. These observations indicated that 2.5% glycerol resulted in better acrosome protection, and 7.8% glycerol gave rise to better motility, progressive motility, BCF, and LIN; therefore, future use of glycerol for cryopreserving chimpanzee sperm should not only focus on evaluating motility-related parameters but also should take into account the assisted reproductive techniques that will later be applied, as sperm head membrane/acrosome integrity is as critically important as motility for successful fertilization upon artificial insemination.

## Conclusions

In this study, we showed that collagenase facilitates the release of chimpanzee spermatozoa from coagulated semen ejaculates, and more importantly we demonstrated for the first time that chimpanzee sperm treated with 0.1% type I collagenase did not alter sperm capacitation status or acrosome integrity. Moreover, using the CASA system, we also showed that sperm motility-related parameters (sperm swimming patterns) were not affected by collagenase treatment. With significant increase of unaltered spermatozoa acquired per ejaculate, we will be able to improve fertilization success and genetic diversity of zoo captive chimpanzee species.

## Additional files

Additional file 1: **Figure S1.** Semen collection from *Pan troglodytes* using artificial vagina. (**A**) Chimpanzee with erected penis (marked with red arrow) ready for semen collection. (**B**) Chimpanzee can tolerate hand message and the use of artificial vagina for semen collection without electroejaculation procedure. Other chimpanzees remained at their designed position in cage without interfering semen collection process. (**C**) Chimpanzee semen was collected from artificial vagina into a 50ml falcon tube using a sterilized scoop (C1); chimpanzee semen coagulated as soon as exposed to the air (C2). (**D**) Positive reinforcement methods were used when semen collection process was completed. (JPEG 1345 kb)

Additional file 2: **Figure S2.** Schematic illustration on experimental design and workflow of this study. (JPEG 172 kb)

## Abbreviations

RT: Room temperature
EEJ: Electroejaculation
PAP: Papanicolaou
PI: Propidium iodine
MTT: (3-(4,5-dimethyl thiazol-2-yl)-2,5-diphenyl tetrazolium bromide)
CASA: Computer-assisted sperm analysis
SEM: Standard error of mean
VAP: Velocity average path
VSL: Velocity straight line
VCL: Velocity curvilinear
ALH: Amplitude of lateral head displacement
BCF: Beat frequency cross
STR: Straightness
PFA: Paraformaldehyde
LDS: Lithium dodecyl sulfate
PVDF: Polyvinylidene Fluoridee
PNA: Lectin peanut agglutinin
