# Supplementary figures and images for: The effects of type I collagenase on the degelification of chimpanzee (Pan troglodytes) semen plug and sperm quality
Source: BMC Vet Res. 2018 Feb 27;14:58. doi: 10.1186/s12917-018-1389-0 (PMC5828100; doi:10.1186/s12917-018-1389-0)

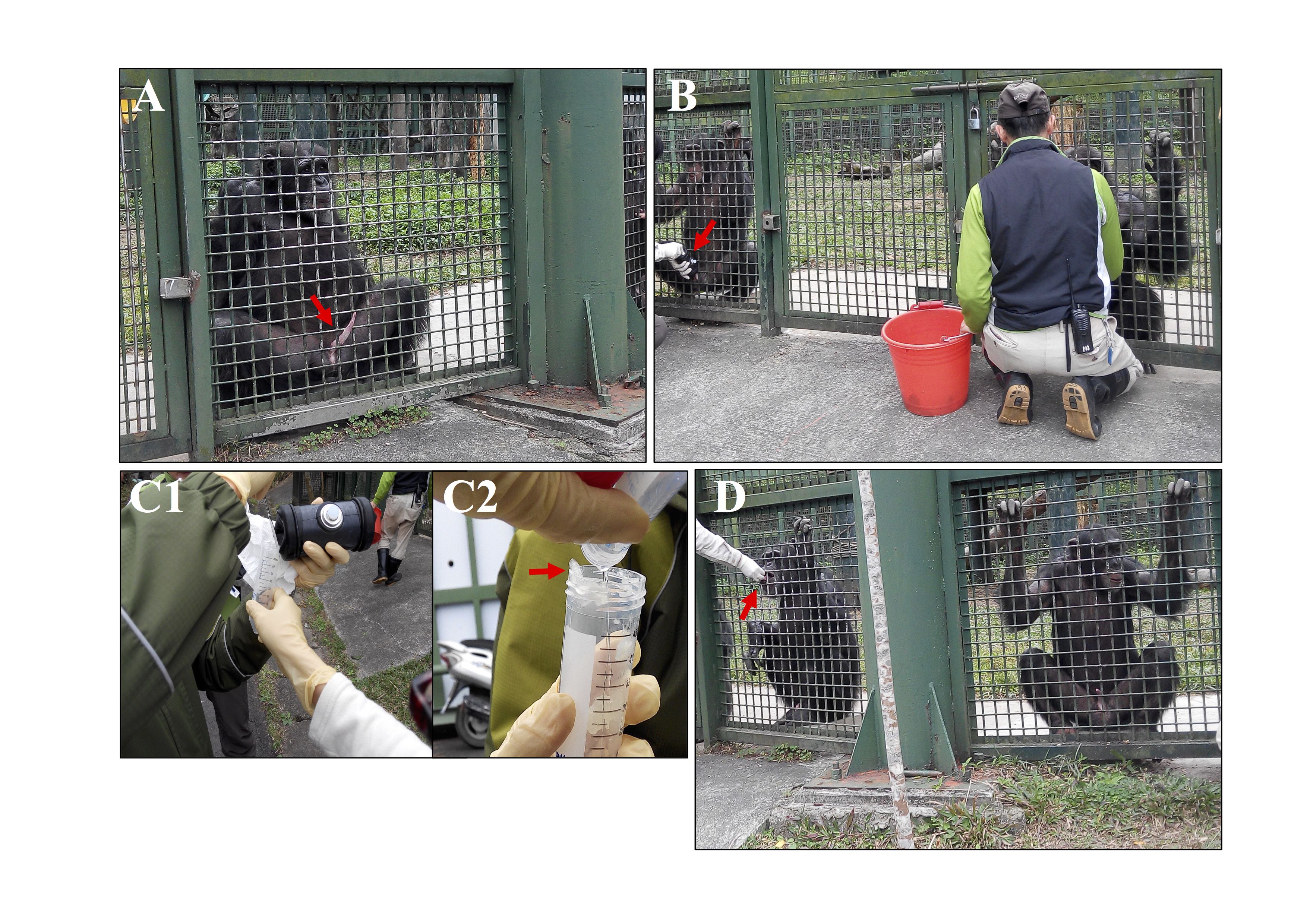

Supplement: Supplementary file 1 — Figure S1. Semen collection from Pan troglodytes using artificial vagina. (A) Chimpanzee with erected penis (marked with red arrow) ready for semen collection. (B) Chimpanzee can tolerate hand message and the use of artificial vagina for semen collection without electroejaculation procedure. Other chimpanzees remained at their designed position in cage without interfering semen collection process. (C) Chimpanzee semen was collected from artificial vagina into a 50ml falcon tube using a sterilized scoop (C1); chimpanzee semen coagulated as soon as exposed to the air (C2). (D) Positive reinforcement methods were used when semen collection process was completed. (JPEG 1345 kb) [file 12917_2018_1389_MOESM1_ESM.jpg]

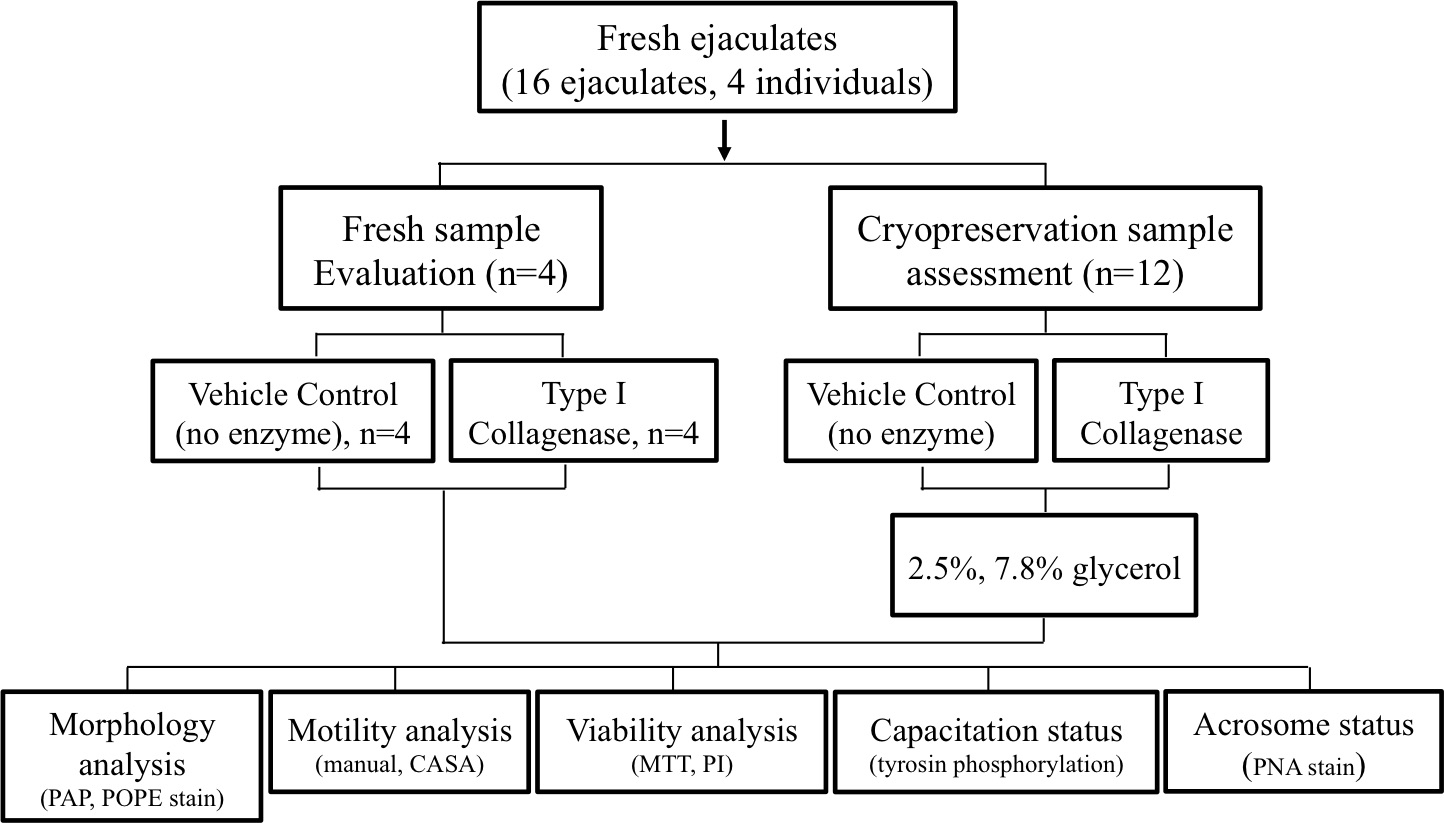

Supplement: Supplementary file 2 — Figure S2. Schematic illustration on experimental design and workflow of this study. (JPEG 172 kb) [file 12917_2018_1389_MOESM2_ESM.jpg]
